# The Application of OCTA in Assessment of Anti-VEGF Therapy for Idiopathic Choroidal Neovascularization

**DOI:** 10.1155/2016/5608250

**Published:** 2016-07-04

**Authors:** Qin Chen, Xiaobing Yu, Zihan Sun, Hong Dai

**Affiliations:** ^1^The Fifth School of Clinical Medicine, Peking University, Beijing 100730, China; ^2^Department of Ophthalmology, Beijing Hospital, Beijing 100730, China

## Abstract

*Purpose*. To assess the morphology of idiopathic choroidal neovascularization (ICNV) by optical coherence tomography angiography (OCTA) and determine the therapeutic effects of intravitreal antivascular endothelial growth factor (anti-VEGF).* Method*. Patients with naive ICNV were assessed by spectral domain optical coherence tomography (SD-OCT) and OCTA in this observational study. The timing of observation was before treatment, 1 day after treatment with intravitreal anti-VEGF injection, and 1 month after the treatment. The central retina thickness (CRT) on SD-OCT, selected CNV area, and flow area on OCTA were measured.* Results*. A total of 17 eyes from 17 patients with ICNV were included in this study. OCTA showed visible irregular choroidal neovascularization with “tree-in-bud” form on outer retinal layer. After treatment, as well as in the 1-day follow-up, CNV decreased in size from the periphery, and the vessel density was reduced. As shown on OCTA, the selected CNV area and flow area were significantly reduced compared to pretreatment. The rate of CNV vessel area changes was higher on OCTA than the changes in CRT on SD-OCT at 1-day and 1-month follow-up.* Conclusion*. Intravitreal injection of anti-VEGF is effective for idiopathic choroidal neovascularization, and the treatment outcomes are observable after 1 day. OCTA provides a useful approach for monitoring and evaluating the treatment of intravitreal anti-VEGF for CNV.

## 1. Introduction

Idiopathic choroidal neovascularization (ICNV) occurs without clear causes in patients younger than 50 years. It is distinct from CNV secondary, pathological myopia, angioid streaks, multifocal choroiditis, punctate inner choroidopathy, presumed ocular histoplasmosis syndrome, and trauma [1, 2]. Choroidal neovascularization (CNV) causes exudation, hemorrhage, and fibrosis at the macula resulting in poor central vision and visual loss.

The pathological basis of ICNV is similar to age-related macular degeneration (AMD). Because vascular endothelial growth factor (VEGF) is part of the pathophysiology of CNV [3], anti-VEGF therapy has become the first-line treatment for ICNV patients [4]. Intravitreal injection of bevacizumab and ranibizumab improves and stabilizes the vision of patient with ICNV [1, 5, 6].

Optical coherence tomography angiography (OCTA) is a new, noninvasive imaging technique that plays an important role in clinical practice [7, 8]. Like ordinary optical coherence tomography (OCT), OCTA provides B-scans of the retina, but OCTA additionally offers rapid, high-resolution, repeated, and accurate visualization of blood flow without injection of a contrast agent, which provides indirect representation of the vascular morphology of the retina and choroid [9].

OCTA has been shown to be effective in the detection of CNV [7, 10]. Lumbroso et al. and Muakkassa et al. used OCTA to detect changes in CNV after anti-VEGF treatment [11, 12]. Furthermore, with recent developments in equipment and software, OCTA can now measure the size of CNV, so that it can be compared before and after anti-VEGF treatment.

Usually, fundus fluorescein angiography (FFA) and OCT are used to evaluate ICNV before and after treatment [6]. No systematic study has been performed on the assessment of ICNV by OCTA. The purpose of our study is to use OCTA to assess the changes in CNV morphology after intravitreal anti-VEGF therapy and compare the results to OCT images.

## 2. Materials and Methods

### 2.1. Subjects

This prospective study was arranged according to the Declaration of Helsinki and approved by the institutional review committee of Beijing Hospital. Informed consent was obtained from all patients. A total of 17 patients of Beijing Hospital with naïve ICNV from May 2015 to January 2016 were included in this study. The diagnosis of ICNV was established in each subject by clinical examination, spectral domain optical coherence tomography (SD-OCT) (RTVue XR AVANTI, Optovue Inc., Fremont, CA) with FFA, and indocyanine green angiography (ICGA) (HRA II, Heidelberg, Germany) when available. Every patient agreed to receive treatment of intravitreal ranibizumab (Lucentis). Every patient was examined three times by SD-OCT and OCTA: the first time was up to three days before treatment, and then the patient was followed 1 day after treatment and 1 month after treatment. To evaluate subretinal and intraretinal fluid, the central retinal thickness (CRT) was measured automatically by SD-OCT.

Exclusion criteria were previous therapy (laser, photodynamic therapy, or anti-VEGF), age-related macular degeneration, myopia, history of trauma, choroiditis, and any other cause of secondary CNV. Eyes that could not be imaged clearly by OCTA and eyes that could not be imaged serially were excluded.

### 2.2. Optical Coherence Tomography Angiography

OCTA images were obtained with an AngioVue OCTA device (RTVue XR AVANTI, Optovue, Fremont, CA, USA). AngioVue uses a split-spectrum amplitude-decorrelation algorithm to detect erythrocyte movement [9]. The macular angiography scan protocol covered a 3 mm × 3 mm area at 840 nm wavelength and 70,000 A-scans per second. The device produced 2 OCT volumes, each consisting of 304 × 304 A-scans with 2 B-scans captured in around 3 seconds. OCTA volumes of the outer retina were automatically segmented by AngioVue software between the Bruch membrane and the inner nuclear layer/outer plexiform layer junction. This region is devoid of blood vessels, so CNV-related abnormal blood flow signals are easy to distinguish. Using AngioAnalytics software, the CNV areas on the outer retina were manually selected by two independent qualified technicians. The selected CNV area values were the data according to the CNV size we chose, and the flow area value was automatically measured as just the detected flow signals within the selected area of CNV.

### 2.3. Statistics

Statistical evaluation was performed using SPSS software (IBM SPSS Statistic 22). All results, including age, gender, best correct visual acuity (BCVA), CRT, selected CNV area, and detected vessels area, were expressed as the mean ± standard deviations. The comparative *t*-test was used to compare CNV changes before and after treatment. To compare lesion changes seen on OCTA with those seen on OCT, the independent-samples *t*-test was used. In all analyses, *P* < 0.05 was considered to be statistically significant.

## 3. Results

This study observed 14 eyes of 14 female patients and 3 eyes of 3 male patients. The mean age of subjects was 34.24 ± 5.68 years. The mean baseline best corrected visual acuity (BCVA) was 59.29 ± 14.80 early treatment diabetic retinopathy study (ETDRS) letters.

**Figure 1:**
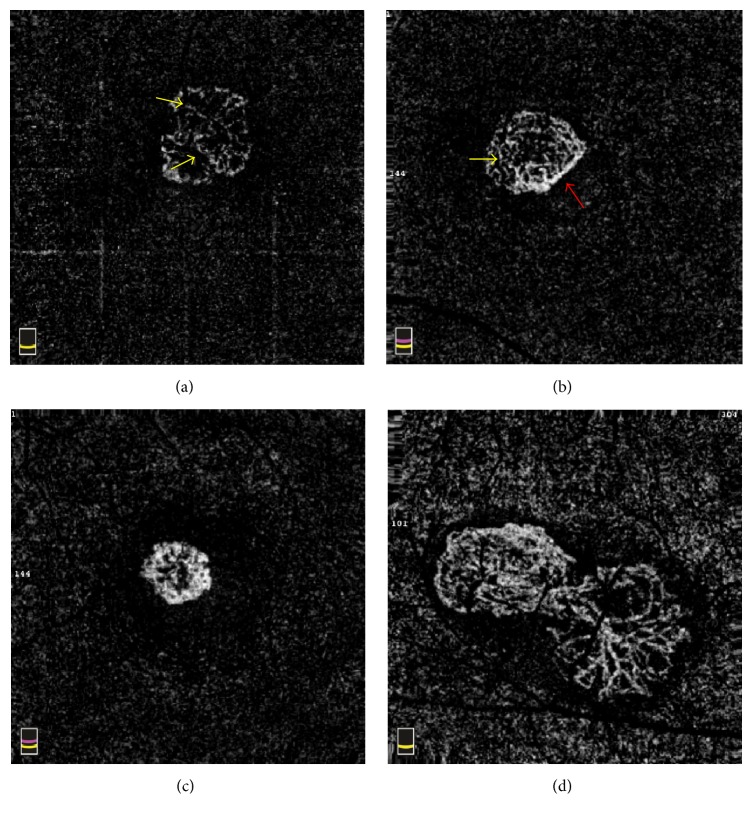
CNV structure of outer retinal layer on OCTA. (a) Irregular circumscribed CNV lesion with abnormal “tree-in-bud” blood flow inside (yellow arrows). (b) A round-like closed CNV with continuous linear peripheral flow (red arrow). (c) An irregular CNV as “medusa-shaped.” (d) The CNV had obvious dendritic branch vessels net from the trunk.

All eyes had pure type 2 choroidal neovascular membranes based on SD-OCT, which showed a highly reflective region that exceeded the retinal pigment epithelium (RPE). Subretinal fluid accumulation around the lesions was seen in 11 eyes and 5 eyes had intraretinal fluid. OCTA presented an irregular or nearly round closed mass at the level of the outer retinal layer in all patients, with convoluted and intertwined vessels. 13 of all had irregular blood flow like small “tree-in-bud” inside lesions (Figure 1(a)). “Tree-in-bud” was defined as a short and discontinuous linearly branching flow signal. OCTA showed that highlighted peripheral flow signals connected to form a continuous line or ring around the vessel mass in 8 eyes (Figure 1(b)). Two eyes had “medusa-shaped” lesions (Figure 1(c)), which were defined as a compact zone of small new blood vessels with minimal internal hypodense structure. One eye had an obvious net of dendritic branch vessels, which branched from the trunk (Figure 1(d)).

**Figure 2:**
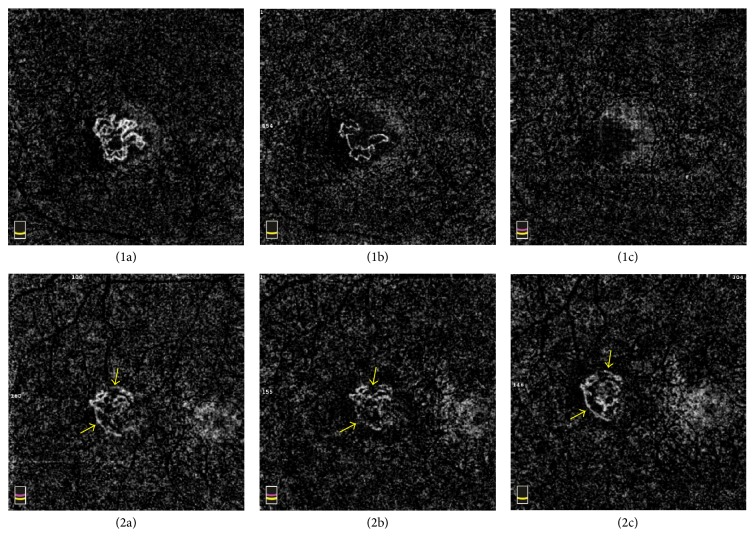
Changes of CNV after intravitreal anti-VEGF treatment of 2 patients 1-2: 3 × 3 OCTA images of each subject. ((a)–(c)) CNVs form before treatment, 1 day after treatment, and 1 month after treatment on OCTA. ((1a)-(1b)) Visible shrinkage of lesion from peripheral vessels and reduction in number and density of vessels at 1 day after treatment. (1c) After 1 month, no detected CNV flow. ((2a)–(2c)) The CNV changes of one female patient whose small abnormal vessels reappeared in previous lesion 1 month after treatment comparing to 1 day (yellow arrows).

All 17 eyes were treated with intravitreal ranibizumab. Follow-up images were obtained 1 day and 1 month after treatment. After 1 day, OCTA showed lesion shrinkage from peripheral vessels in 16 eyes and reduction in the number and density of vessels. Small vessels narrowed and fragmented. In some case, peripheral blood flow decreased and became filamentous. The average selected CNV area and flow area were significantly smaller than those before treatment (*t* = 4.559, *P* < 0.01; *t* = 5.775, *P* < 0.01). However, intraretinal and subretinal fluid as imaged by SD-OCT did not change or showed only a small amount of reduction. One month later, the mean BCVA for all patients increased by 7 letters to 66.11 ± 10.86 ETDRS letters (*t* = −1.533, *P* = 0.135). SD-OCT showed reduction of retinal fluid. On the OCT angiogram, 14 of 17 eyes showed a continued decrease of visible vessels and CNV area size and in 1 eye the CNV had completely disappeared (Figure 2(1a–1c)). The vessel reduced with pruning of thinner peripheral flow. The network density decreased, but some vessels with large diameters were still retained. The selected CNV area and flow area became smaller than those before treatment (*t* = 4.182, *P* < 0.01; *t* = 4.249, *P* < 0.01) and were smaller than 1 day after treatment (*t* = 3.591, *P* < 0.01; *t* = 2.823, *P* < 0.05), and the difference was statistically significant (Table 1). However, in 3 eyes, OCTA showed that the flow area increased, comparing to 1-day follow-up. The small vessels inside the previous lesion and some peripheral vessels reappeared (Figure 2(2a–2c)).

**Figure 3:**
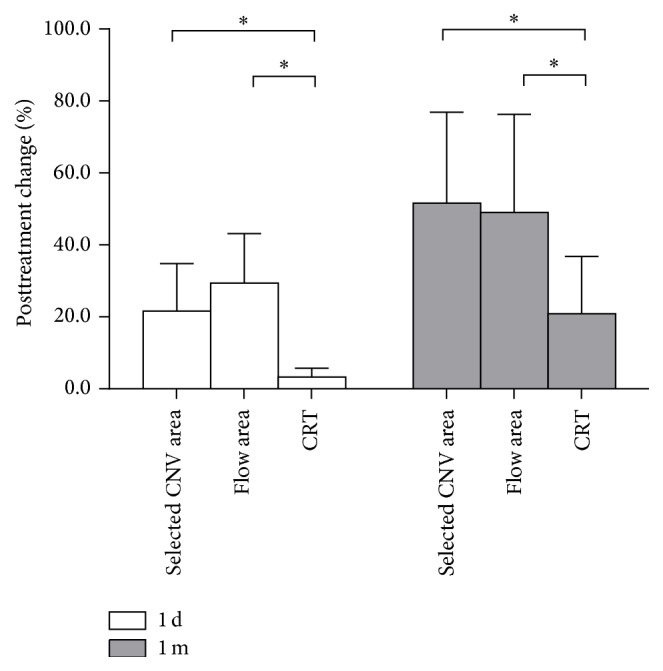
Comparison of 1-day and 1-month posttreatment changes on selected CNV area, flow area, and CRT. ^*∗*^ *P* value < 0.01.

During every visit, CRT on SD-OCT was also measured. The selected CNV area and flow area were reduced by 21.65% and 29.41%, respectively, compared to a 3.29% decrease in CRT 1 day after treatment. This was a larger, significant difference (*t* = 5.651, *P* < 0.01; *t* = 7.732, *P* < 0.01). Although there were signs of recurrence in 3 patients with CNV at the 1-month visit, on average, there was a 51.65% decrease in the selected CNV areas and a 49.00% decrease in the flow areas, which was significantly larger than the 20.88% decrease in CRT (*t* = 4.256, *P* < 0.01; *t* = 3.674, *P* < 0.01) (Figure 3).

**Figure 4:**
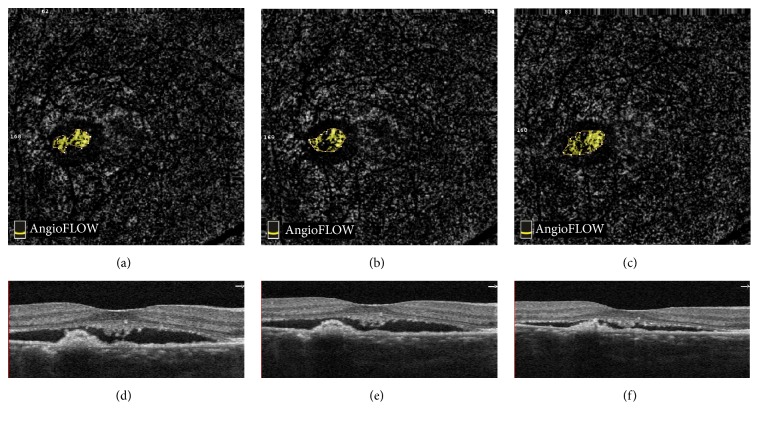
OCTA images with associated B-cans of a female patient. ((a)–(c)) CNVs form before treatment, 1 day after treatment, and 1 month after treatment on OCTA. The selected CNV areas were 0.089 mm^2^, 0.097 mm^2^, and 0.116 mm^2^. The flow areas were 0.048 mm^2^, 0.045 mm^2^, and 0.060 mm^2^. ((d)–(f)) B-scans of 3 detections. The CRT was 367 *μ*m, 354 *μ*m, and 287 *μ*m. The subretinal fluids were decreased continuously.

One eye showed opposing results with OCTA versus OCT. OCTA indicated that the patient's CNV area was continuously increased after intravitreal treatment at the 1-day and 1-month follow-ups. BCVA was also reduced by 18 EDTRS letters. However, the subretinal fluid seemed to be absorbed and CRT dropped from 367 *μ*m to 287 *μ*m (Figure 4).

## 4. Discussion

Currently, in clinical situations, FFA and ICGA are the golden standards for detecting CNV [13, 14]. Both methods are invasive examinations that require injections and can cause nausea, vomiting, and, rarely, anaphylaxis. In contrast, OCTA is noninvasive and does not require dye injection. OCTA is a new technology that has provided layered angiograms of retinal and choroidal blood vessels. An OCTA-enabled method is called split-spectrum amplitude-decorrelation angiography (SSADA), which detects motion in the blood vessel lumen by measuring the variation in reflected OCT signal amplitude between consecutive cross-sectional scans. Moreover, OCTA is sensitive to observing the size and shape of CNV without obscured details due to dye leakage, as occurs with FFA and ICGA.

In ICNV patients, OCTA showed the CNV structures to be varied, mostly irregular, or nearly round closed masses with internal arborescent capillaries. This confirms some reports that OCTA visualized CNV as irregular, closely knit flow formations at the level of the outer retinal layer [15, 16]. Most abnormal vessels of ICNV had “tree-in-bud” blood flow. We hypothesized that CNVs of ICNV are composed of many tightly coiled blood vessels, where the blood flowing through multiple turns or knotted parts is likely to form turbulence. However, OCT-A measures only the linear blood flow [9]. Turbulent motion could present as a dark area, which impeded the conversion and representation of this flow on a hyperdense frame. That may be why there were short and discontinuous linear branching flow signals.

The “tree-in-bud” structure is different from the large central trunk-like or “sea fan” vessels in AMD [17–19]. In this study, about 11% of the eyes (2/17) had “medusa-shaped” lesions, and the rate of this structure was lower than those in AMD patients [20]. Previous trials proved that ICNV was treated more effectively by anti-VEGF drugs than AMD, because the former needed fewer injections and gained better vision [6, 21]. Although the size of the lesion and duration of disease were related to treatment effects, we speculate that different neovascular morphology, such as “tree-in-bud” in ICNV and other structures in AMD, will cause different outcomes and prognosis after anti-VEGF therapy.

Some eyes were found to have highlighted peripheral blood flow around the CNV membrane. It was indicated that these lesions might have an anastomotic vessel bounding the outer border of the vascular lesions, which have played an important role in the pathophysiology of CNV [18]. After the treatment, CNV in most eyes continued to shrink and microvascular flow was rarefaction, although large internal vessels persisted, which was mentioned in another study [17]. This implies that anti-VEGF treatment is more effective in causing the vanishing of small vessels than large, mature vessels. The sensitivity of smaller capillaries to anti-VEGF therapy may be associated with the mechanism of VEGF in angiogenesis [22, 23]. We believed that the decrease of vessels density was mainly due to the loss of smaller capillaries [24], which were not visible because of collapse or very slow flow. For 3 of 17 eyes in this study, the OCTA images showed that small internal vessels reappeared 1 month after treatment, and CNV flow areas increased compared to the 1-day posttreatment exam. This suggested that the patients should be treated once again. Since OCTA can be repeated often while FFA or ICGA should not be, OCTA can be a better way to monitor CNV development after anti-VEGF treatment. In the future, more research with OCTA is needed to find the timing for retreatment on OCTA.

Currently, SD-OCT is used regularly in evaluating clinical effect of anti-VEGF treatment in patients with CNV. OCT examination provides valuable data to confirm the characteristics of CNV, especially intraretinal and subretinal fluid, pigment epithelium detachment, and central retinal thickness [25]. As a noninvasive examination, OCT is essential at every visit after the treatment. Nowadays, OCTA is a useful tool to detect CNV area and flow. Muakkassa et al. reported that the area and greatest linear dimension (GLD) of CNV were simple measurements to quantify the CNV size after treatment [12]. Jia et al. also used quantitative measurements of CNV flow and area to evaluate treatment effects in neovascular AMD [26].

With the clinical application of OCTA, the assessments of treatment for CNV can be more developed and diverse. In this study, selected CNV area and detected vessel flow were measured with analysis software that was developed for OCTA, and the responses to treatment were basically consistent with OCT images and ETDRS letter exams. In addition, the rates of change of CNV size and detected vessel flow as shown on OCTA were higher than the CRT change on SD-OCT. One day after intravitreal injection, there were no obvious changes of fluid on OCT, but the selected CNV area and flow area were significantly smaller than those before treatment. The mean rate of changes on OCT and on OCTA presented a larger significant difference between the two detection methods. This study showed that the selected CNV area and flow area could be more sensitive for evaluating treatment outcomes, instead of relying solely on visual acuity and OCT B-scans. Additionally, the quantitative analysis confirmed that intravitreal ranibizumab is an effective treatment for ICNV. In the future, CNV size and vessel area in ICNV patients will be examined regularly to monitor the response to treatment, especially in early stage after treatment.

In 1 of the 17 eyes, it was found that the selected CNV area and flow area were increased after treatment, and BCVA decreased. Although these changes indicated that the condition of CNV had worsened, the OCT image showed reduction of subretinal fluid and CRT. In another one eye, OCTA showed that the CNV vessel area decreased with increased CRT value, and the BCVA improved after treatment. This suggested that OCTA performance did not correspond to OCT in some cases but did correspond to vision changes.

Retina fluid is caused by the increase of vascular permeability and leakage, so subretinal fluid and the CRT value on SD-OCT do not accurately reflect CNV structure or abnormal blood flow. OCTA might better reflect the state of CNV, because it can detect the vessel flow directly. Future studies are needed to assess the two measurements for monitoring the therapeutic effect of ICNV.

There were several limitations to this study. Firstly, this observational study had a limited number of patients, and patients with poor fixation or imaging quality were excluded, which may have caused selection bias. Secondly, the follow-up period after treatment was short, which only included 1 day and 1 month. Additional observation phases might help to explain the atrophy and reperfusion of CNV vessels. Moreover, the selected CNV area was performed manually. Although trained and qualified personnel drew every area twice, the possibility of measurement error existed. With OCTA, SSADA software can only detects blood flow in a certain range of speeds [9, 15]. The blood flow is too slow or too fast, such vessels will not be displayed in the OCTA. Therefore, OCTA could not completely reflect the changes of vessels in this study. CNV was observed and detected only at the outer retinal layer that was usually avascular in the background, which did not accurately reflect CNV vessels overall. In the future, improved software for three-dimensional volumetric analysis would be an ideal measurement for quantifying CNV lesion.

## 5. Conclusion

In summary, OCTA enables the observation of abnormal vascular morphology of ICNV from the horizontal section, examines changes of CNV after anti-VEGF treatment, and quantitatively analyzes variation in selected CNV areas or flow areas with specific analysis software. By using OCTA as an assessment method, intravitreal injection of ranibizumab was shown to be effective for idiopathic choroidal neovascularization, and CNV shrinkage from the periphery as well as vessel density reduction was observable after 1 day of treatment. OCTA provides a useful approach for monitoring and evaluating the treatment effects of intravitreal anti-VEGF for ICNV.

## Figures and Tables

**Table 1:** Comparison of pretreatment and posttreatment measurements of ICNV.

	Pretreatment	1-day posttreatment	1-month posttreatment	*t*			*P* value		
BCVA letter (*n*)	59.29 ± 14.80	—	66.11 ± 10.86	−1.533^*∗∗∗*^			0.135^*∗∗∗*^		
Selected CNV area (mm^2^)	0.41 ± 0.43	0.33 ± 0.38	0.19 ± 0.25	4.559^*∗*^	3.591^*∗∗*^	4.182^*∗∗∗*^	<0.01^*∗*^	<0.01^*∗∗*^	<0.01^*∗∗∗*^
Flow area (mm^2^)	0.19 ± 0.19	0.14 ± 0.17	0.09 ± 0.11	5.775^*∗*^	2.832^*∗∗*^	4.249^*∗∗∗*^	<0.01^*∗*^	<0.05^*∗∗*^	<0.01^*∗∗∗*^

BCVA, best corrected visual acuity; CNV, choroidal neovascularization; ^*∗*^Comparing pretreatment to 1-day posttreatment measurements; ^*∗∗*^Comparing 1-day to 1-month posttreatment measurements; ^*∗∗∗*^Comparing pretreatment to 1-month posttreatment measurements.

## References

[1] Qi H.-J., Li X.-X., Tao Y. (2010). Outcome of intravitreal bevacizumab for idiopathic choroidal neovascularization in a Chinese population. *Canadian Journal of Ophthalmology*.

[2] Shah S. N., Kang Q. Y., Fan X. J., Shahbaz F. (2015). Microstructural effects of intravitreal bevacizumab in idiopathic choroidal neovascularisation. *Journal of Ayub Medical College, Abbottabad*.

[3] Campochiaro P. A. (2013). Ocular neovascularization. *Journal of Molecular Medicine*.

[4] Jian L., Panpan Y., Wen X. (2013). Current choroidal neovascularization treatment. *Ophthalmologica*.

[5] Cheema R. A., Mushtaq J., Cheema M. A. (2011). Intravitreal bevacizumab as a primary treatment for idiopathic choroidal neovascularization. *Middle East African Journal of Ophthalmology*.

[6] Carreño E., Moutray T., Fotis K. (2015). Phase IIb clinical trial of ranibizumab for the treatment of uveitic and idiopathic choroidal neovascular membranes. *British Journal of Ophthalmology*.

[7] de Carlo T. E., Romano A., Waheed N. K., Duker J. S. (2015). A review of optical coherence tomography angiography (OCTA). *International Journal of Retina and Vitreous*.

[8] Jia Y., Bailey S. T., Hwang T. S. (2015). Quantitative optical coherence tomography angiography of vascular abnormalities in the living human eye. *Proceedings of the National Academy of Sciences of the United States of America*.

[9] Jia Y., Tan O., Tokayer J. (2012). Split-spectrum amplitude-decorrelation angiography with optical coherence tomography. *Optics Express*.

[10] Mastropasqua R., Di Antonio L., Di Staso S. (2015). Optical coherence tomography angiography in retinal vascular diseases and choroidal neovascularization. *Journal of Ophthalmology*.

[11] Lumbroso B., Rispoli M., Savastano M. C. (2015). Longitudinal optical coherence tomography-angiography study of type 2 naive choroidal neovascularization early response after treatment. *Retina*.

[12] Muakkassa N. W., Chin A. T., De Carlo T. (2015). Characterizing the effect of anti-vascular endothelial growth factor therapy on treatment-naive choroidal neovascularization using optical coherence tomography angiography. *Retina*.

[13] Yannuzzi L. A. (2011). Indocyanine green angiography: a perspective on use in the clinical setting. *American Journal of Ophthalmology*.

[14] Wu A. M., Wu C. M., Young B. K., Wu D. J., Margo C. E., Greenberg P. B. (2015). Critical appraisal of clinical practice guidelines for age-related macular degeneration. *Journal of Ophthalmology*.

[15] Lumbroso B., Huang D., Jia Y., Fujimoto J. G., Rispoli M. (2015). *Clinical Guide to Angio-OCT: Non Invasive, Dyeless OCT Angiography*.

[16] Wang Q., Chan S. Y., Jonas J. B., Wei W. B. (2016). Optical coherence tomography angiography in idiopathic choroidal neovascularization. *Acta Ophthalmologica*.

[17] Kuehlewein L., Sadda S. R., Sarraf D. (2015). OCT angiography and sequential quantitative analysis of type 2 neovascularization after ranibizumab therapy. *Eye (Basingstoke)*.

[18] Spaide R. F. (2015). Optical coherence tomography angiography signs of vascular abnormalization with antiangiogenic therapy for choroidal neovascularization. *American Journal of Ophthalmology*.

[19] Kuehlewein L., Bansal M., Lenis T. L. (2015). Optical coherence tomography angiography of type 1 neovascularization in age-related macular degeneration. *American Journal of Ophthalmology*.

[20] El Ameen A., Cohen S. Y., Semoun O. (2015). Type 2 neovascularization secondary to age-related macular degeneration imaged by optical coherence tomography angiography. *Retina*.

[21] Razi F., Haq A., Tonne P., Logendran M. (2016). Three-year follow-up of ranibizumab treatment of wet age-related macular degeneration: influence of baseline visual acuity and injection frequency on visual outcomes. *Clinical Ophthalmology*.

[22] Cui J. Z., Kimura H., Spee C., Thumann G., Hinton D. R., Ryan S. J. (2000). Natural history of choroidal neovascularization induced by vascular endothelial growth factor in the primate. *Graefe's Archive for Clinical and Experimental Ophthalmology*.

[23] Byrne A. M., Bouchier-Hayes D. J., Harmey J. H. (2005). Angiogenic and cell survival functions of Vascular Endothelial Growth Factor (VEGF). *Journal of Cellular and Molecular Medicine*.

[24] Lumbroso B., Rispoli M., Savastano M. C., Jia Y., Tan O., Huang D. (2016). Optical coherence tomography angiography study of choroidal neovascularization early response after treatment. *Developments in Ophthalmology*.

[25] Coscas F., Coscas G., Souied E., Tick S., Soubrane G. (2007). Optical coherence tomography identification of occult choroidal neovascularization in age-related macular degeneration. *American Journal of Ophthalmology*.

[26] Jia Y., Bailey S. T., Wilson D. J. (2014). Quantitative optical coherence tomography angiography of choroidal neovascularization in age-related macular degeneration. *Ophthalmology*.

